# European survey on CAR T-Cell analytical methods from apheresis to post-infusion immunomonitoring

**DOI:** 10.3389/fimmu.2025.1567582

**Published:** 2025-04-24

**Authors:** Biagio De Angelis, Maria Luisa D’Amore, Pacôme Lecot, Kerstin Neininger, Margot Lorrain, Laetitia Gambotti, Caroline Dreuillet, Elise Courcault, Sampurna Chatterjee, Julio Delgado, Anne Galy, Paul Franz, Juan Roberto Rodriguez-Madoz, Yolanda Cabrerizo, Anne Richter, Charis Girvalaki, Maddalena Noviello, Elena Tassi, Carmen Sanges, Maik Luu, Michael Hudecek, Andreas Kremer, Franco Locatelli, Helene Negre, Concetta Quintarelli

**Affiliations:** ^1^ Department of Oncology-Haematology, and Cell and Gene Therapy, Bambino Gesù Children’s Hospital, IRCCS, Rome, Italy; ^2^ Department of Clinical Research, Institute National du Cancer (French National Cancer Institute-INCa), Boulogne-Billancourt, France; ^3^ Information Technology for Translational Medicine S.A., Esch-sur-Alzette, Luxembourg; ^4^ Takeda Development Center Americas, Inc., Lexington, MA, United States; ^5^ Takeda Pharmaceuticals U.S.A., Inc., Lexington, MA, United States; ^6^ Hospital Clinic Barcelona, Insitut de Investigacions Biomediques August Pi i Sunyer, Barcelona, Spain; ^7^ Accelerator of Technological Research in Genomic Therapy (ART-TG), US35, Inserm, Corbeil-Essonnes, France; ^8^ Fraunhofer Institute for Cell Therapy and Immunology IZI, Leipzig, Germany; ^9^ Hemato-Oncology Program, Cima Universidad de Navarra, IdiSNA, Pamplona, Spain; ^10^ European Hematology Association (EHA), Den Haag, Netherlands; ^11^ Miltenyi Biotec GmbH, Bergisch Gladbach, Germany; ^12^ European Cancer Patient Coalition, Brussels, Belgium; ^13^ Division of Immunology, Transplantation, and Infectious Diseases, Experimental Hematology Unit, IRCCS San Raffaele Scientific Institute, Milan, Italy; ^14^ Lehrstuhl für Zelluläre Immuntherapie, Medizinische Klinik und Poliklinik II, Universitätsklinikum Würzburg, Würzburg, Germany; ^15^ Department of Pediatrics, Catholic University of the Sacred Heart, Rome, Italy; ^16^ Institut de Recherche et Développement Servier Paris-Saclay, Gif-sur-Yvette, France; ^17^ Department of Clinical Medicine and Surgery, University of Naples Federico II, Naples, Italy

**Keywords:** European survey, CAR T-cells, T2Evolve, immunomonitoring, lymphodepleting chemotherapy, leukapheresis

## Abstract

**Background:**

Chimeric Antigen Receptor (CAR) T-cell therapy has emerged as a revolutionary approach to cancer treatment. Given the rapid expansion of new indications addressed by newly developed CAR T-cell products, it is essential to standardize analytical methods for the characterization/monitoring of apheresis materials, drug products, and post-infusion patient samples.

**Methods:**

The T2Evolve Consortium, part of the European Union's Innovative Medicines Initiative (IMI), conducted an extensive anonymous online survey between February and June 2022. Comprising 36 questions, the survey targeted a wide range of stakeholders involved in engineered T-cell therapies, including researchers, manufacturers, and clinicians. Its goal was to address the current variability within the CAR T-cell field, focusing on analytical assays for quality control of apheresis materials, drug products, and post-infusion immunomonitoring. Another objective was to identify gaps and needs in the field.

**Results:**

A total of 53 respondents from 13 european countries completed the survey, providing insights into the most commonly used assays for apheresis material and drug product characterization, alongside safety and efficacy tests required by the Pharmacopeia. Notably, a minority of respondents conducted phenotypical characterization of T-cell subsets in the drug product and assessed activation/exhaustion T cell profiles.

**Conclusion:**

The survey underscored the necessity to standardize CAR T-cell functional potency assays and identify predictive biomarkers for response, relapse, and toxicity. Additionally, responses indicated significant variability in CAR T-cell monitoring during short-term patient follow-up across clinical centers. This European survey represents the first initiative to report current approaches in different stages of CAR T-cell therapies via a survey, from drug product quality controls to post-infusion immunomonitoring. Based on these findings, and with input from T2EVOLVE experts, the next step will be to address harmonization in the identified areas. These efforts are anticipated to significantly enhance cancer patients' access to engineered T cell therapy safely and effectively throughout Europe.

## Introduction

In recent years, the groundbreaking application of genetically modified T cells using Chimeric Antigen Receptor (CAR) has revolutionized the treatment landscape of patients with B cell acute lymphoblastic leukemia (B-ALL) or B-cell non-Hodgkin lymphoma, as well as with multiple myeloma. This innovation has led to the market approval of seven products by the U.S. Food and Drug Administration (FDA), with the most recent being obecabtagene autoleucel, and six products by the European Medicines Agency (EMA) (1–7).

Several studies, both academic and company-driven, are exploring the use of CAR T cells in different other indications, including relapsed/refractory T-cell acute lymphoblastic leukemia (T-ALL)/T cell lymphoblastic lymphoma (T-LBL) (8), acute myeloid leukemia (AML) (9, 10), and solid tumors (11–15).

Recent impressive data have also emerged regarding the use of anti-CD19 CAR T-cell therapy for the treatment of selected non-malignant diseases, namely systemic lupus erythematosus (SLE) (16–18), Myasthenia gravis (19) and other autoimmune conditions (20), taking into account the role played by autoreactive B cells in its pathogenesis (21, 22).

Considering the emerging worldwide scenario of new CAR T-cell development and clinical trials, it could be anticipated that a significant number of these innovative CARs will soon reach the approval by regulatory bodies.

However, to enable comparison of the clinical data collected in the current trials and in the outcoming ones, it is essential to standardize the characterization of the leukapheresis material, CAR T-cell drug products and patient’s immunomonitoring. Specifically, there is an urgent need to reach a global consensus on quality control assays to be performed for in-process controls (during manufacturing process) and for the characterization and release of the drug product (23). In addition, it is also necessary to harmonize and standardize the methods and timing of immunomonitoring of patients treated with CAR T cells across different involved laboratories, and hopefully, provide a rationale for pre-selecting groups of patients with high probability of benefiting from CAR T cell infusion.

In 2021, the T2EVOLVE consortium was established under the European Union’s Innovative Medicines Initiative (IMI) with the aim of aiding Europe in expediting the development of engineered T-cells and enhancing patient access to innovative medical treatments (https://t2evolve.com/). Among the objectives of T2EVOLVE, there is the harmonization of analytical methods employed for evaluating the quality of leukapheresis, characterizing drug products, and monitoring the immune response of patient’s post-infusion.

In 2022, T2EVOLVE launched an anonymous online survey to comprehensively capture the diverse pre- and post-infusion CAR T analytical assays conducted across Europe. It sought to identify gaps and requirements for enhancing the comparability of clinical trials and standardizing quality controls. The survey was inclusive, welcoming participation from a broad spectrum of stakeholders, including contract research organizations (CROs)/contract manufacturing organizations (CMOs), physicians, biologists, biotechnologists, pharmacists, leukapheresis centers, and experts in patient immunomonitoring.

The outcomes of the survey lay the groundwork for a prioritized list of analytical methods aimed at standardizing procedures, particularly focusing on the starting material and drug products. Notably, the survey responses underscored the urgency of standardizing CAR T-cell potency assays and identifying predictive biomarkers for response, relapse, and toxicity. Additionally, the findings highlighted substantial variability in the practices of CAR T-cell monitoring during short-term patient follow-up across different clinical centers. The valuable insights obtained from the survey are instrumental in steering global initiatives towards advancing standardization, promoting comparability, and ultimately improving the overall efficacy within the field of engineered T-cell therapies.

## Methods

### Survey platform

The start-up “Information Technology for Translational Medicine” (ITTM), a partner of the T2Evolve, developed a digital health web interface for conducting an anonymous survey. The T2Evolve survey content was prepared by the T2Evolve experts participating to the WP5 (a project working package dedicated to “Gold standard analytical methods in manufacture and monitoring of CAR T cells”), and was then shared across the whole consortium to be validated for its comprehensiveness and applicability.

### Survey design

The survey consisted of 36 questions (including both multiple choice and open-ended questions) distributed across six sections (**Supplementary Tables S1**-**2**) and related to: 1) demographic information (**Supplementary Table S2 section A**), 2) apheresis product collection and quality control procedures (**Supplementary Table S2 section B**), 3) quality control assays for raw materials used in engineered T cell manufacturing (**Supplementary Table S2 section C**), 4) in-process and release quality control assays for the drug product (**Supplementary Table S2 section D**), 5) lympho-depletion regimen (**Supplementary Table S2 section E**), and 6) post-infusion immunomonitoring (**Supplementary Table S2 section F**). Although queries were suitable for both autologous and allogeneic settings, participants exclusively reported experiences with autologous products. Thus, we focused the analysis solely on autologous CAR T-cell products.

### Survey diffusion

The survey was specifically targeted at European stakeholders actively involved in the field of CAR T-cell immunotherapy. The primary audience included biotech, CROs, CMOs, as well as academic institutions, including public and private hospitals, leukapheresis centers, GMP facilities, development labs, and immunomonitoring units. The survey was launched in early February 2022 and concluded at the end of June 2022. It was distributed via a web survey link and quick-response (QR) code provided through the T2Evolve website, and further disseminated through international scientific conferences and email campaigns. Responses were monitored biweekly throughout the five-month period.

### Survey analysis and statistics

The anonymous replies were analyzed using in-house downstream analysis pipelines based on Jupyter Notebook and Python programming language (version: python#3). All anonymous responses are available in Comma-Separated Values (CSV) and JavaScript Object Notation (JSON) text format, with the latter serving as the input format for the downstream analysis pipeline. Results were expressed as an absolute number of respondents or as a percentage of respondents among all participants who answered the given question.

To ensure the validity of the survey results, stringent acceptability criteria were implemented to minimize the risk of representational bias among respondents. Specifically, a maximum acceptability threshold of 5% was enforced for responses received from the same organization within a single country. However, this restriction was not applied when the same organization participated across different countries. No minimum number of respondents was required to represent a country. Each respondent was uniquely identified by three key elements: country, city, and organization name. For the evaluation, only surveys that were at least 50% completed were considered valid, and responses were assessed for consistency with the questions posed.

## Results

### Demographic information of responding centers

**Figure 1:**
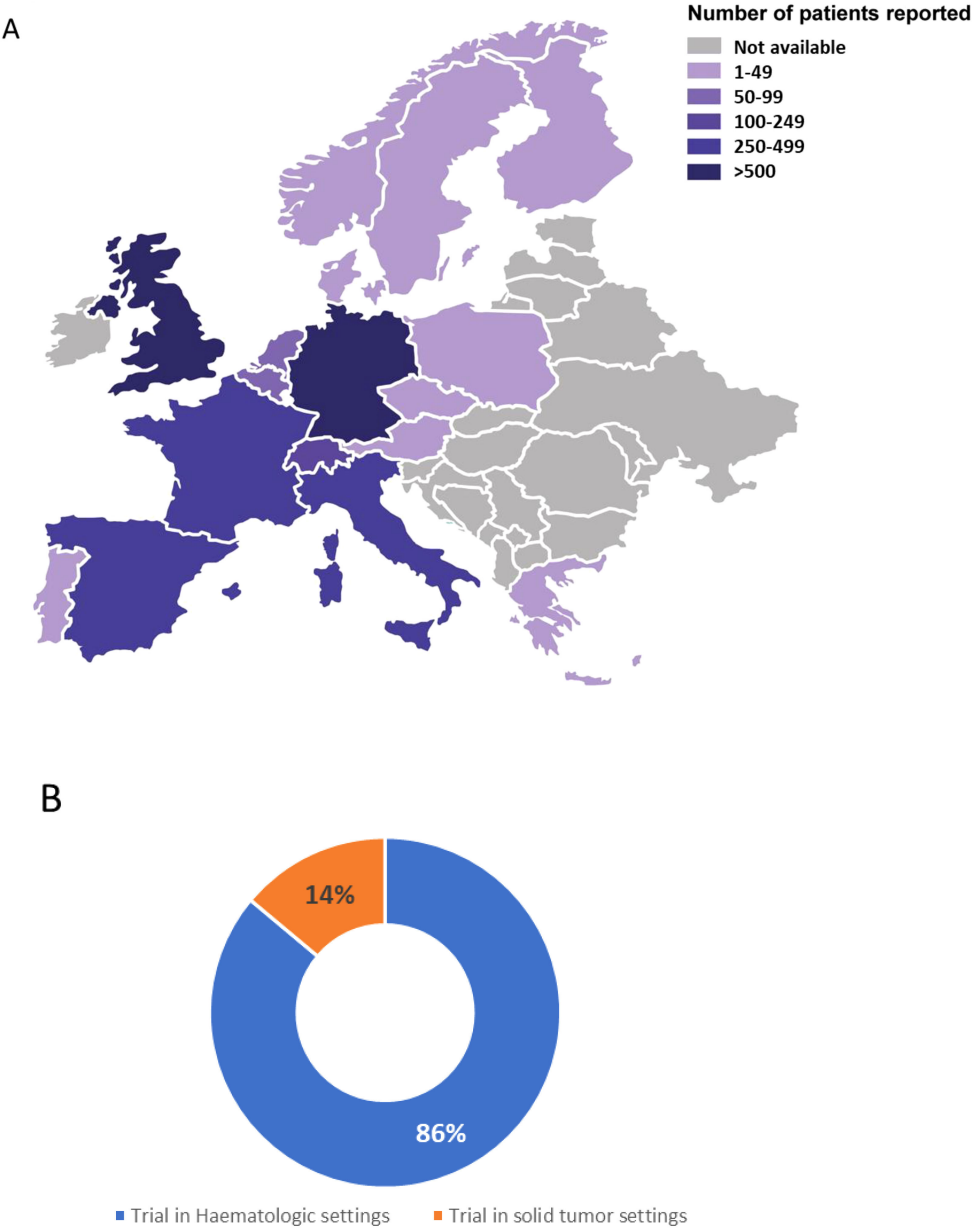
Distribution of CAR T-cell treated patients across Europe. **(A)** Map representing the distribution of CAR T-cell treated patients per country in Europe based on the EBMT Registry (as of March 2022, i.e. at the time of the survey distribution). Number of treated patients is reflected by the color intensity. **(B)** Distribution of recruiting or active clinical trials as of the end of the survey in June 2022, sourced from ClinicalTrials.gov, in the field of CAR T-cell therapy for the treatment of hematologic indications (including ALL, lymphoma, multiple myeloma, T-ALL, and AML) and solid tumor indications (such as renal carcinoma, neuroblastoma, sarcoma, and unspecified metastatic advanced solid cancers).

In 2021, more than 3000 patients had been treated with CAR T-cell therapy in Europe. By comparison, more than twice the number of patients (6,343) were treated in the United States (US). Additionally, in the same year, 16% of patients in Europe and 14% in the US received CAR T-cell therapy in the context of a clinical trial. Regarding geographic distribution, the European Society for Blood and Marrow Transplantation (EBMT) Registry, reported that the access to CAR T-cell therapy is higher in Western Europe than in Eastern Europe in 2022 (**Figure 1A**, EBMT website). This scenario appears to align with the distribution of respondents in the current survey, with the exception of UK and Spain, for which only one and three survey participants, respectively, were recorded. Moreover, data from the ClinicalTrials.gov platform indicate that, at the time of the survey, there were 36 interventional clinical trials in Europe related to CAR T therapy, with 86% focusing on hematologic indications (including ALL, lymphoma, multiple myeloma, T-ALL, and AML) and 14% targeting solid tumor indications (such as renal carcinoma, neuroblastoma, sarcoma, and undefined metastatic advanced solid cancers) (**Figure 1B**).

The T2EVOLVE Consortium launched a European anonymized online survey consisting of 36 questions, and covering all the most relevant topics related to CAR T-cell therapy.

A total of 58 participants took part in the survey, including 53 from Europe, 4 from the United States, and 1 from Israel. Since the dissemination plan primarily targeted European countries, the response rates from the US and Israel were deemed too low to accurately represent these countries’ involvement in the field. As a result, respondents from the US and Israel were excluded from the analysis.

**Figure 2:**
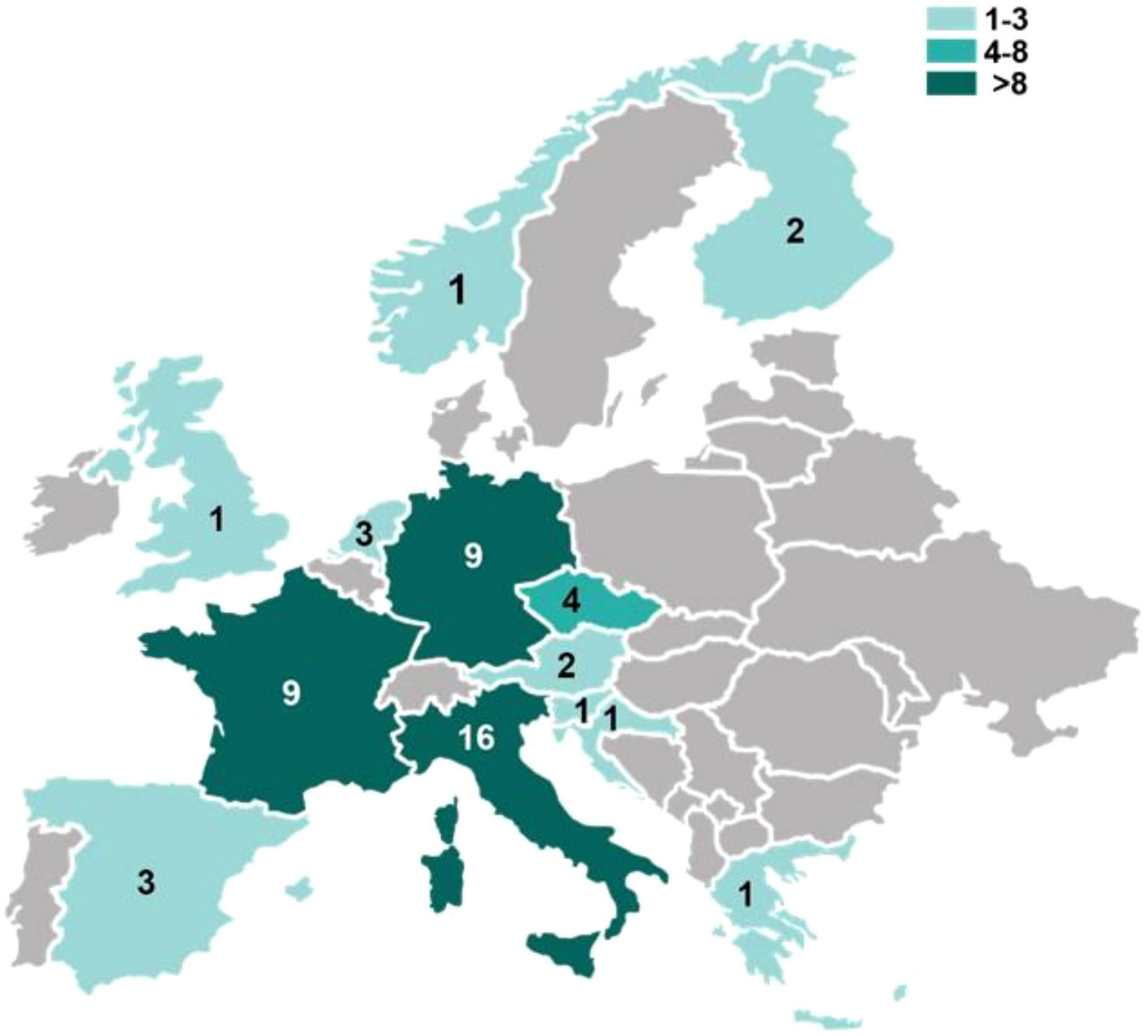
Distribution of T2Evolve survey responding centers across Europe. Map representing the number of the survey responding centers per country in Europe. Number of respondents is reflected by the color intensity.

The 53 European participants who responded to the T2EVOLVE survey represented 13 European countries. The majority of responses came from Italy (30%, n=16), France (17%, n=9), and Germany (17%, n=9) (**Figure 2**, **Supplementary Table S3**). Among the Italian and French respondents, more than half was concentrated in Rome (9 out of 16) and Paris (6 out of 9), respectively, while respondents from other countries were more evenly distributed (**Supplementary Table S3**).

A significant majority of respondents (77%, n=41) were affiliated with either public or private hospitals, while 17% (n=9) represented pharmaceutical companies (**Table 1**). Additionally, 2 respondents (4%) were from CROs or CMOs, and one respondent (2%) was from an applied research organization. In terms of professional role, most respondents were clinicians (42%, n=22), followed by preclinical researchers (30%, n=16) and GMP manufacturing operators (26%, n=14) (**Table 1**).

**Table 1:** Survey respondents’ affiliation.

Type of organization of respondents		Number of respondents	% of respondents among all
Public/Private hospital		41	77.4
Pharmaceutical Biotech Company		9	17.0
Contract Research Organization/Contract Manufacturing Organization		2	3.8
Applied Research Organization		1	1.9
**Type of field/department of respondents**		Number of respondents	% of respondents among all
Preclinical	Cell & Gene Therapy Unit	7	13.2
	Research & Development	6	11.3
	Immunology, Laboratory, Hematology	3	5.7
GMP	GMP Facility	9	17.0
	Quality Control	3	5.7
	Quality Assurance	2	3.8
Clinical	Clinical (oncology, pediatric, hematology, other…) AND Stem Cell/Transfusion/Transplantation department	22	41.5
	Clinical Research (clinical trial management	1	1.9

Number and percentage of respondents who reported to be allocated to a given type of organization, field and department.

**Figure 3:**
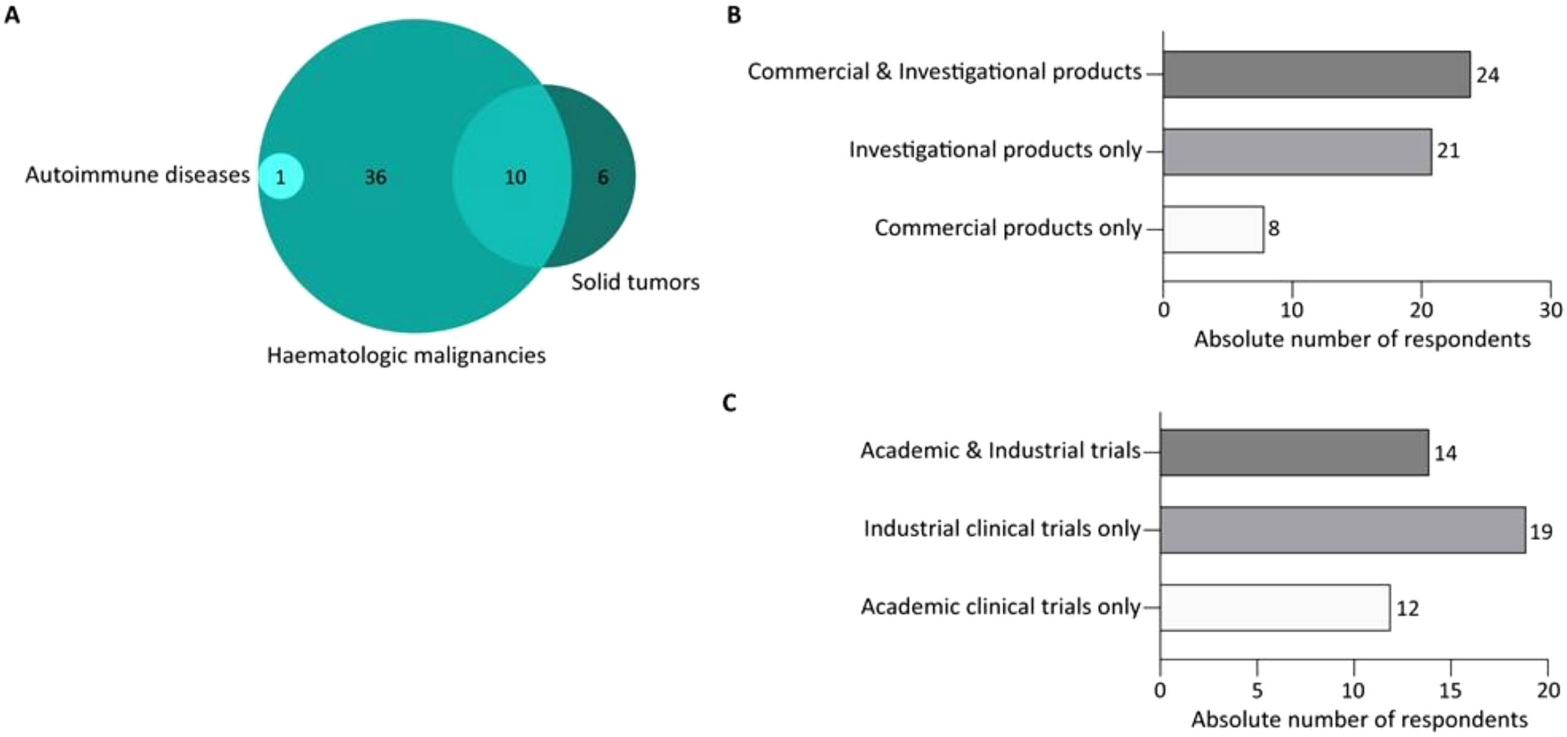
CAR T-cell application expertise for the survey respondents. **(A)** Venn diagram representing the number of respondents and their expertise in the field of CAR T-cells for hematological malignancies, and/or in solid tumors, and/or in auto-immune diseases. **(B)** Bar graph representing the number of respondents divided for their expertise in the use of commercial and/or investigational CAR T-cell products. **(C)** Bar graph representing the number of respondents divided for their involvement in context of academic or/and industrial-driven clinical trials.

The majority of responding centers declared their involvement in the development or administration of CAR T-cells for hematologic malignancies (89%, n=47) (**Figure 3A**), while only 30% (n=16) reported use of CAR T-cells for solid tumors. Notably, 19% of participants (n=10) indicated that their centers were involved in both hematologic and solid tumor therapies. Additionally, 1 center reported using CAR T-cells for the treatment of autoimmune diseases. Interestingly, 45% of respondents (24 out of 53) reported to work with both commercial and investigational CAR T-cells (**Figure 3B**). Among the 45 respondents working with investigational CAR T-cells, 27% of centers (12 out of 45) were exclusively involved in academic clinical trials (**Figure 3C**). The survey questions did not enable the recording of additional details regarding the CAR T-cell product type or the specific indications for which it is used at the respondents’ sites.

### Quality controls of the apheresis product intended for the manufacture of autologous CAR T-cell drug product

**Figure 4:**
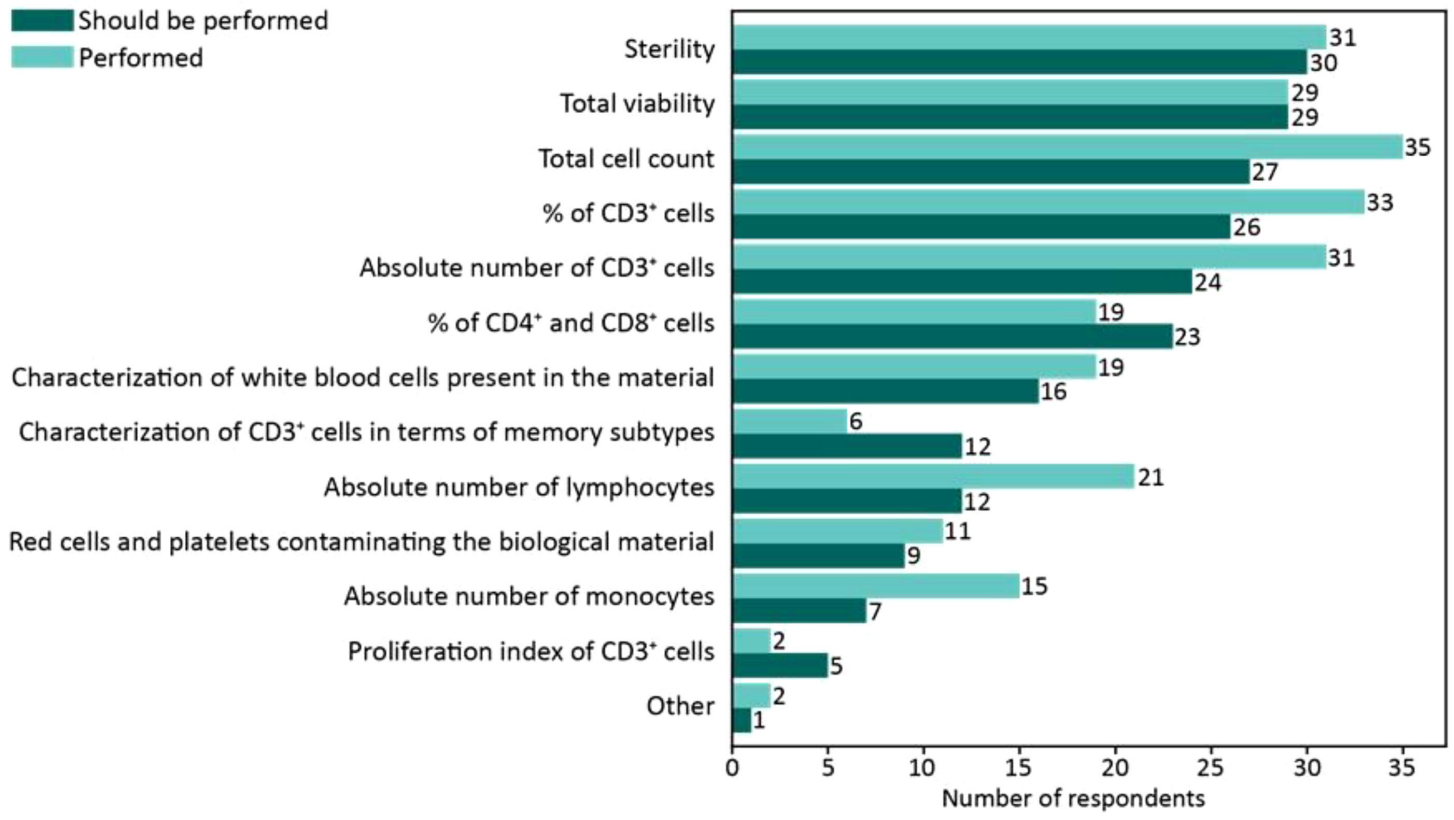
Survey responses regarding analytical assays performed on the apheresis product. The bar chart illustrates the number of respondents performing the specified analytical assays on the apheresis product. The lighter-colored bars represent the frequency of assays currently performed, while the darker-colored bars indicate the number of respondents who believe these assays should be performed to optimize leukapheresis characterization. The data highlight the gap between current practices and perceived needs for improving apheresis product analysis.

Among the 53 respondents, 72% (38 out of 53) indicated that they routinely perform quality control assays for apheresis products. Of these, 76% (29 out of 38) reported conducting sterility tests, viability assays, total cell counts, and assessments of the percentage and absolute count of T-lymphocytes (CD3+ cells) (**Figure 4**).

In response to an open-ended question regarding improvements needed in the analytical control of apheresis products to standardize practices, 35 respondents highlighted the importance of standardizing both the apheresis process and its associated quality controls. Indeed, they specifically emphasize the importance of standardizing the characterization of the apheresis via flow cytometry. In addition, respondents call for greater consensus on the protocol for apheresis collection in case of cryopreservation and the identification of predictive biomarkers associated with successful manufacturing.

### CAR T-cell manufacturing and release

In the context of CAR T-cell manufacturing process, we did not capture the scenario of centers that use only commercial setting, so it is luckily that the data provided in this section refer to the manufacturing of academic products, or of industrial investigational products in early clinical trial development. All respondents reported using viral vector-transduced CAR T-cells, specifically retroviral or lentiviral vectors. Regarding the raw materials, 75% (30 out of 40) of participants in this survey section, reports that they did not perform any analytical tests on the raw materials, relying solely on the certificate of analysis provided by the suppliers.

**Figure 5:**
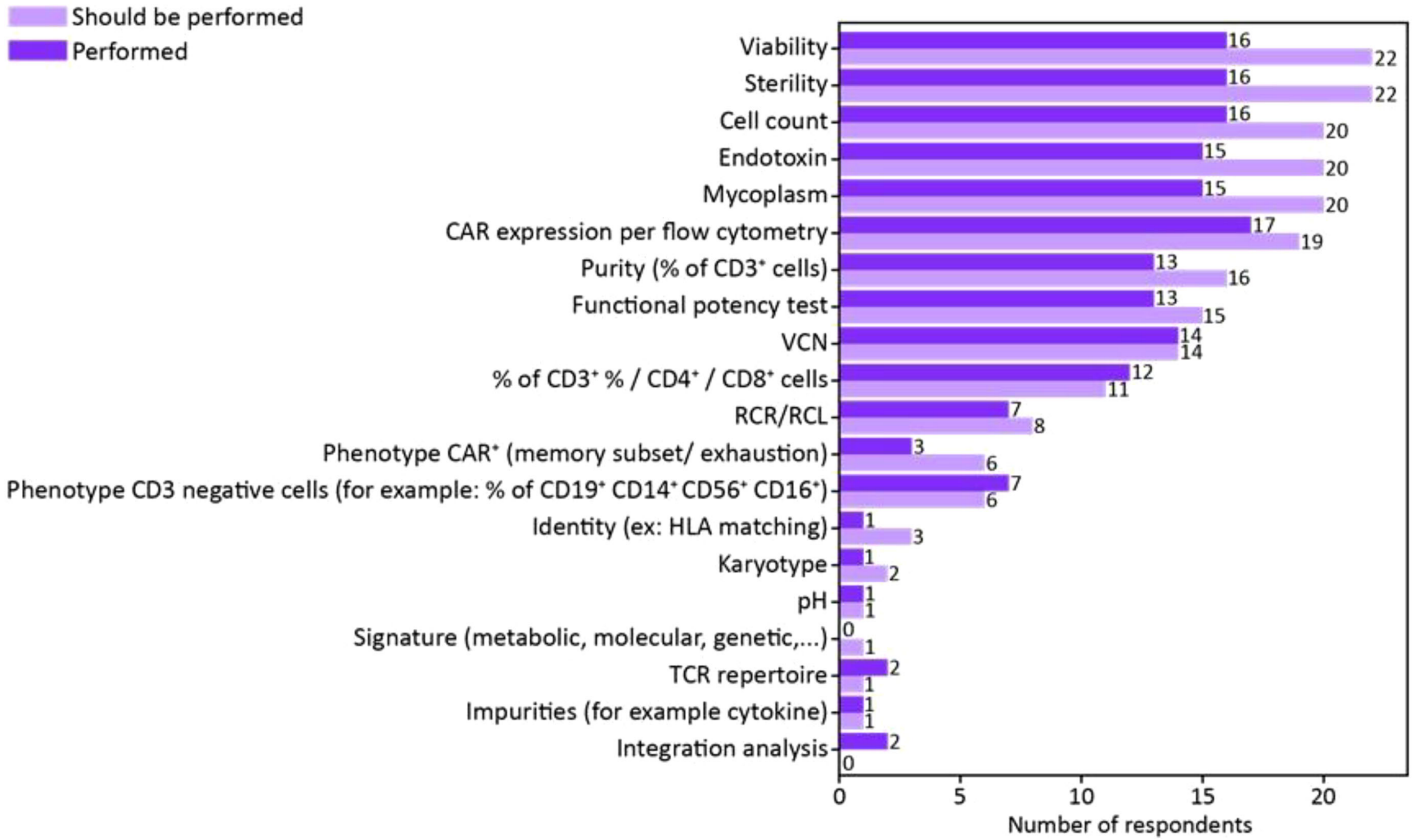
Survey responses regarding analytical assays performed to release CAR T-cell drug product. The bar chart illustrates the number of respondents performing the specified analytical assays on the drug product. The darker-colored bars represent the frequency of assays currently performed, while the lighter-colored bars indicate the number of respondents who believe these assays should be performed to optimize drug product characterization. The data highlight the gap between current practices and perceived needs for improving drug product analysis.

When it comes to the analytical methods used for the release and characterization of autologous CAR T-cell drug products, only 34% of survey participants (18 out of 53) were directly involved in the manufacturing or release of an engineered autologous T-cell product and provided details on the assays routinely performed. Among these respondents, at least 83% (15 out of 18) conducts safety and identity tests, including viability, sterility, cell count, endotoxin, mycoplasma, and CAR expression by flow cytometry, all in accordance with the European Pharmacopeia and Good Manufacturing Practice (GMP) guidelines. However, only a small portion (16.67%; 3 out of 18) carries out an in-depth characterization of the drug product, focusing on T-cell subsets and activation/exhaustion profiles (**Figure 5**), and no one of the Respondents is performing integration site analysis.

In response to the open-ended question, regarding the major needs in terms of standardization in the release of engineered T-cell products, 21 respondents emphasize the necessity of establishing gold-standard methods for assays assessing replication-competent lentivirus (RCL) or replication-competent retrovirus (RCR), vector copy number (VCN), potency, and drug product characterization by flow cytometry panels.

Additionally, respondents highlighted the positive impact of developing and validating rapid sterility assays to shorten the release time of autologous CAR T-cell products. They also stressed the importance of identifying and implementing predictive biomarkers closely associated with patient response and potential toxicity.

### Lymphodepletion regimens for patients undergoing CAR T-cell infusion

Regarding lymphodepletion (LD) regimens prior to CAR T-cell infusion, 100% (29 out of 29) of participants report using fludarabine and cyclophosphamide (Flu/Cy) as part of pre-conditioning protocols. Additionally, 3 out of 29 respondents also use bendamustine (of the three centers, one declared the use of only academic products, whereas a second one of industrial CAR T cells in investigational trial), while 2 respondents (one of which declaring the use of only academic products) do not perform LD in patients who are already cytopenic at time of treatment. Lastly, two respondents—possibly from the same institution, as they indicated being from the same city and exclusively infusing industrial CAR T-cells in early development—reported using a different lymphodepletion regimen involving Alemtuzumab.

For the characterization of patient samples collected before LD, 56% of respondents (20 out of 36) reports performing immunophenotyping of peripheral blood leukocytes, 31% (11 out of 36) assesses the expression of the CAR target antigen on tumor cells, and 36% respondents (13 out of 36) does not conduct any analysis prior to LD. Notably, 1 respondent monitors cytokine levels in patient samples before LD.

After LD but before CAR T-cell infusion, 53% of respondents (20 out of 38) reports performing immunophenotyping of leukocytes and lymphocytes in peripheral blood, 24% (9 out of 38) monitored cytokine levels, and 42% (16 out of 38) does not carry out any analysis following LD.

In response to the open-ended question regarding the major needs in terms of standardization of assays in pre- and post- LD timing, 33 out of 38 respondents (86.84%) emphasize the need for consensus on biological markers associated with toxicity. They also underscore the necessity for standardized guidelines on the optimal LD regimen, including the choice of chemotherapy drugs and their respective doses.

### Immunomonitoring of patients following CAR T-cell infusion

Lastly, the survey included 13 additional questions focused on the types of immunomonitoring performed after CAR T-cell infusion by clinical centers across Europe. In particular, 97% (28 out of 29) of respondents in this survey section, reported their experience with CAR T-cell monitoring, while only 3% (1 out of 29) had experience with other engineered T cells, namely T cells engineered with a specific T Cell Receptor (TCR).

The first set of questions aimed to determine the most common methods used for monitoring the persistence of CAR T-cells and the assays used to characterize these cells. The majority of respondents (27 out of 29; 94%) reported using flow cytometry to evaluate the persistence of infused CAR T-cells, regardless the use of commercial or investigational drug products in the academic or industrial setting. Additionally, 45% (13 out of 29) utilizes molecular assessments, including real-time PCR (25%) and digital droplet PCR (ddPCR) (16%). Notably, 34% (10 out of 29) conducts both molecular and flow cytometric evaluations. While 77% of participants limits their quantitative assessments to peripheral blood, 23% extends their analyses to other samples, such as bone marrow, cerebrospinal fluid, lymph nodes, and tumor tissues. Most respondents characterize engineered T cells in detail, focusing on CD4+/CD8+ subsets (80%), memory cell profiles (67%), exhaustion status (60%), activation status (50%), and cytokine production (37%). Interestingly this characterization is performed also by centers that declare the solely use of commercial products. Fewer respondents perform integration analysis (17%), TCR repertoire analysis (17%), or ex-vivo functional assays (13%).

Given the risk for severe toxicity events associated with CAR T-cell therapy, such as cytokine release syndrome (CRS) and immune effector cell-associated neurotoxicity syndrome (ICANS), early detection and appropriate management are crucial. To this end, 41% of respondents reported conducting additional tests to monitor for CRS and/or ICANS, while 33% retains biological samples for future analysis without performing immediate additional tests. The remaining 26% does not collect or analyze further samples.

In cases of tumor relapse, 52% of respondents undertake additional analytical measures to characterize relapsed tumors, 28% keeps samples for future analysis, and 21% neither conduct further analyses nor collect additional samples.

Regarding potential anti-CAR immunity, which can affect the persistence and efficacy of engineered T cells, only 13% (3 out of 23) of respondents performs tests for Human Anti-Mouse Antibodies (HAMA).

**Figure 6:**
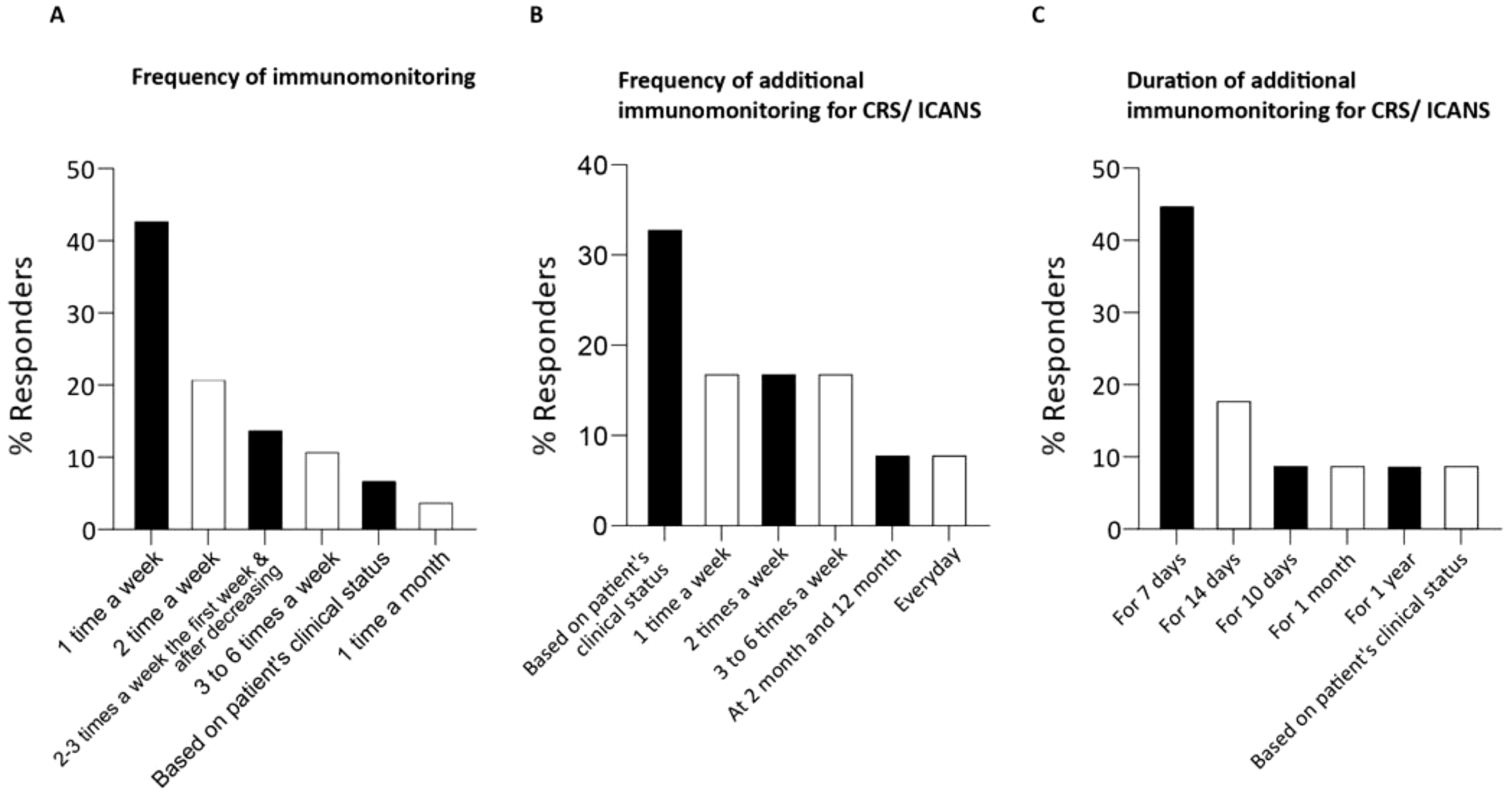
Survey responses regarding immunomonitoring schedule of patients following CAR T-cell infusion. **(A)** The bar chart illustrates the frequency of immunomonitoring performed by the respondents. Percentage of respondents has been calculated on the 29 total respondents for this survey section. **(B)** The bar chart illustrates the frequency of immunomonitoring performed by the respondents following the occurrence of CRS/ICANS/other toxicities (after infusion of CART cells). **(C)** The bar chart illustrates the duration of immunomonitoring performed by the respondents following the occurrence of CRS/ICANS/other toxicities. Percentage of respondents has been calculated on the 11 total respondents for this survey section.

The frequency of the CAR T-cells monitoring significantly varied among respondents, with 43% checking infused CAR T-cells once a week, 21% twice a week, and 11% 3 to 6 times a week. The remaining 25% conducts monitoring sporadically or on clinician demand (**Figure 6A**). The length of the immunomonitoring period across respondents suggests a lack of consensus, ranging from two weeks to years after the CAR T-cell infusion.

Half of the respondents (48%, 12 out of 25) performed additional monitoring following CAR T-cell infusion to investigate CRS/ICANS or other toxicities. The frequency and duration of monitoring for these toxicities varied significantly: 33% adjusts monitoring based on the patient’s clinical status (**Figure 6B**), 8% performs daily blood sampling, 17% monitors 3 to 6 times a week, 17% twice a week, and another 17% once a week. Most respondents monitor patients for 7 to 14 days (**Figure 6C**), though 27% extends monitoring up to one year or based it on the patient’s condition.

When asked about improvements for immunomonitoring in the first 12 months post-infusion, 38 out of 53 respondents (71.7%) emphasize the need to identify and validate specific biomarkers to predict clinical outcomes, toxicity, and early relapse. They also advocate for proficiency testing programs to ensure consistent pharmacokinetic results across laboratories. Some respondents suggest implementing longitudinal sampling and deep immune phenotyping (of apheresis products and blood pre- and post-CAR T-cell infusion) using flow cytometry and single-cell RNA sequencing (scRNAseq). Additionally, they recommend establishing a general flow-cytometry antibody panel to include markers for exhaustion, activation, and differentiation on CAR T-cells, as well as evaluating changes in non-CAR cell populations to monitor bystander effects.

## Discussion

T2Evolve, an alliance of academic and industry leaders in cancer immunotherapy under the European Union’s IMI, has the goal of accelerating the development and expanding access to immunotherapies involving T cells genetically engineered with CARs or TCRs for cancer patients.

To gain insights into immunotherapy practices in Europe and support the harmonization of analytical methods for assessing leukapheresis quality, characterizing drug product, and post-infusion patient immuno-monitoring, T2Evolve conducted an anonymized online survey between February and June 2022. The survey aimed to provide a comprehensive overview of the current state of CAR T-cell therapy in Europe. Despite being open for only five months, the survey garnered 58 responses, 53 of which from European centers. As expected, most responses originated from EU countries with high volumes of CAR T-cell treatments, namely Italy, Germany, and France. In contrast, despite their active role in the field, the UK and Spain showed lower participation in the survey. Notably, CAR T-cell administration is not evenly distributed within individual countries, with the majority of patients being treated in a limited number of highly specialized centers. This uneven distribution makes it challenging to fully address the potential geographical bias inherent in this European survey. Nevertheless, the coverage of the survey in terms of treated indications aligned with the expected one, as for the data retrieved from clinicaltrial.gov showing that in Europe, at the time of the survey, 86% of the trials were recruiting/active to infuse CAR T cells in patients with hematologic disease. The vein-to-vein process of autologous CAR T-cell manufacturing, which is governed by strict laws and regulations, starts with the collection of the patient’s leukapheresis (24, 25). Although leukapheresis is a critical step in ensuring the quality and success of CAR T-cell production, various protocols are currently employed to collect sufficient T cells for drug product manufacturing (26). Previous studies have highlighted that efficient mononuclear cell collection via apheresis, which preserves T-cell cytotoxic functions, is a critical step in the CAR T-cell manufacturing process (25, 27). This survey confirms the importance of this critical aspect.

Several studies have highlighted various factors that may influence the efficiency of T-cell collection for CAR T-cell manufacturing (27). Efforts have also been made to identify predictors of the total number of target cells collected. Despite these studies and the numerous ongoing clinical trials, consensus protocols for apheresis collection remain limited, and phenotypic and functional characterizations are inconsistently applied. Indeed, the survey revealed that only a few centers using commercial CAR T-cell products conduct a characterization of leukapheresis products in terms of white blood cells, red blood cells, and platelets. In contrast, such analyses are more commonly performed at centers administering investigational products, whether from academic or industrial sources. This variability can lead to inconsistent product quality, potentially affecting therapeutic outcomes.

Among the manipulations performed on apheresis collections, cryopreservation offers significant advantages. It provides scheduling flexibility, enabling leukapheresis to be conducted when the patient’s health is optimal for obtaining higher-quality T cells. Cryopreservation also mitigates the impact of shipping delays and removes time constraints before manufacturing, as noted by Tyagarajan et al. (28)

While there have been discussions about standardizing protocols for cryopreservation (e.g., ensuring the process is completed quickly, ideally within 24 hours of collection), no global consensus has been established. The need to harmonize practices to ensure that every patient receives a product of consistently high quality, regardless of where or when the apheresis takes place, have been confirmed also in this survey, since 66% of respondents emphasized the need for better standardization of the apheresis process and cryopreservation procedures.

Additionally, there is a growing demand for the identification of specific biomarkers associated to the aphaeresic product that could predict the success of the manufacturing. Biomarkers could provide early indicators of cell yield and quality, enabling clinicians to optimize the apheresis procedure on a per-patient basis. This would be particularly beneficial in tailoring the procedure to individual patients, potentially improving the efficiency of CAR T-cell manufacturing and enhancing clinical outcomes (29, 30).

Moving on following steps of CAR T-cell manufacturing, leukapheresis is transferred to a GMP-facility, where CAR T-cells are generated by viral or non-viral transduction (31). Regarding T-cell transduction, all survey’s respondents indicated that the production of CAR T-cells for the drug products used in their experience, is based on viral transduction, and that retroviral or lentiviral vectors are applied.

Prior to release for clinical use, CAR T-cell drug products have to address specific release criteria confirming that product’s identity, purity, safety, and effectiveness align with required standards (32). While no significant differences in the quality control methods applied to drug products were reported, 52% of respondents emphasized the need for establishing standardized methodologies for release assays, including the quantification of RCR/RCL, determining Vector Copy Number, conducting potency assays, and implementing standardized flow cytometry panels for CAR T-cell characterization. The use of standardized release tests would allow comparison between drug products produced in different centers, facilitating the comparison of data generated across drug products and centers/countries.

The production of CAR T-cells requires rigorous quality control evaluations to monitor the entire process and ensure that the final product is safe and effective for patient administration. However, when dealing with fresh CAR T-cell drug product that need to be infused into patients shortly after production, or when implementing an accelerated production timeline (ranging from 14 days to just a few days) (33), quality control testing can present significant challenges. These challenges stem from the time-consuming nature of traditional quality control tests, which may hinder the ability to adapt the production process in real-time (34). To address this issue, there is a strong emphasis on developing faster quality control methods, such as rapid sterility tests, to ensure the safety and quality of CAR T-cell products before they are administered to patients. Implementing these faster testing procedures would help streamline the production process, reducing the vein to vein time, while maintaining the high standards necessary for clinical use.

LD regimens has been identified as a critical factor in the success of CAR T-cell treatment (35), because it 1) reduces patient’s lymphocytes to allow CAR T-cell expansion, 2) prevents CAR T-cell depletion through tumor cell reduction 3) reprograms the microenvironment after CAR T-cell infusion (36).

Data reported in the literature currently consider the combination of Flu and Cy to be the most commonly used LD regimen (37). Most survey participants confirmed the use of this regimen in Europe, for both the commercial and the investigational setting, and the hematologic and solid tumors indications. Indeed, current lymphodepletion regimens are primarily based on experience maturated in hematologic malignancies, which have driven the clinical development of CAR T-cell therapy. However, clinical data on the impact of lymphodepletion for CAR T-cell therapy in solid tumors are limited. These regimens should be specifically tailored for solid tumors to address the unique changes in the tumor microenvironment (TME), a factor that differs from hematologic malignancies. For example, Srivastava et al. demonstrated in a xenograft model of lung adenocarcinoma that incorporating oxaliplatin into the Cy lymphodepletion regimen, administered prior to CAR-T cell infusion, activates lung tumor macrophages to produce T cell-recruiting chemokines. This approach leads to enhanced CAR-T cell infiltration, remodeling of the TME, and increased tumor sensitivity to anti-PD-L1 (38). In line with this consideration, the majority of the participants underlined the need to define the best LD regimes based on the patient profile/disease, able to generate a favorable cytokine milieu, but preventing hematological and non-hematological toxicities, as well as infections. Recently, Ghilardi et al, reported that in refractory or relapsed large B-cell lymphomas, bendamustine for LD before tisagenlecleucel has similar efficacy to Flu/Cy with reduced toxicities, including CRS, ICANS, infectious and hematological toxicities, as well as reduced hospital utilization (39). The survey captured the use of bendamustine for patient conditioning in three participating centers. Despite broad consensus on the use of LD prior to CAR T-cell therapy, respondents also emphasized the need to standardize pre-infusion immuno-monitoring and optimize LD regimens. This includes establishing consistent timing, which typically occurs within a week before CAR T-cell infusion, ensuring at least two resting days to mitigate any potential negative impact of chemotherapy (40), as well as standardizing the type and doses of chemotherapy for different cancer types.

CAR T-cells are ‘living drugs’ with an unpredictable ability to expand *in vivo* that changes from one patient to another. For this reason, the recent best practices of EBMT and JACIE for the management of CAR T-cell therapy recommend close monitoring of medium- and long-term CAR T- cell persistence (41).

In post-infusion immunomonitoring, CAR T-cell persistence can be assessed using various methods, depending on the CAR T-cell product, the knowledge of the CAR genome sequence, and the availability of appropriate trackable markers (42). The commercial availabilities of appropriate antigen-based (43) and antibody-based (idiotypic) (44) detection methods have enabled the study of CAR(+) T-cells both before and after their adoptive transfer. The survey revealed high variability in immuno-monitoring practices, including sample types, timing, and duration, making it challenging to compare persistence data across studies. Among the different techniques, the large number of respondents conducts immunomonitoring by flow-cytometry; however, also ddPCR and real-time PCR have been used. This finding is in line with the current literature that considers real-time PCR and flow cytometry as the most valuable assays for longitudinal monitoring of CAR T-cells, as they are well established and widely available (45, 46). Moreover, Berger et al, demonstrated the correlation between results obtained by ddPCR and flow-cytometry, emphasizing the importance of using complementary assays for more accurate evaluation (47).

A notable finding was that less than half of respondents conducted additional monitoring in case of occurrence of CRS, ICANS, or other post-infusion toxicities. This highlights a lack of consensus on specific biomarkers for managing CRS/ICANS, despite evidence linking soluble biomarkers like IL-6 (48), IFN-γ (49) and IL-1 (50) to toxicity grading.

**Figure 7:**
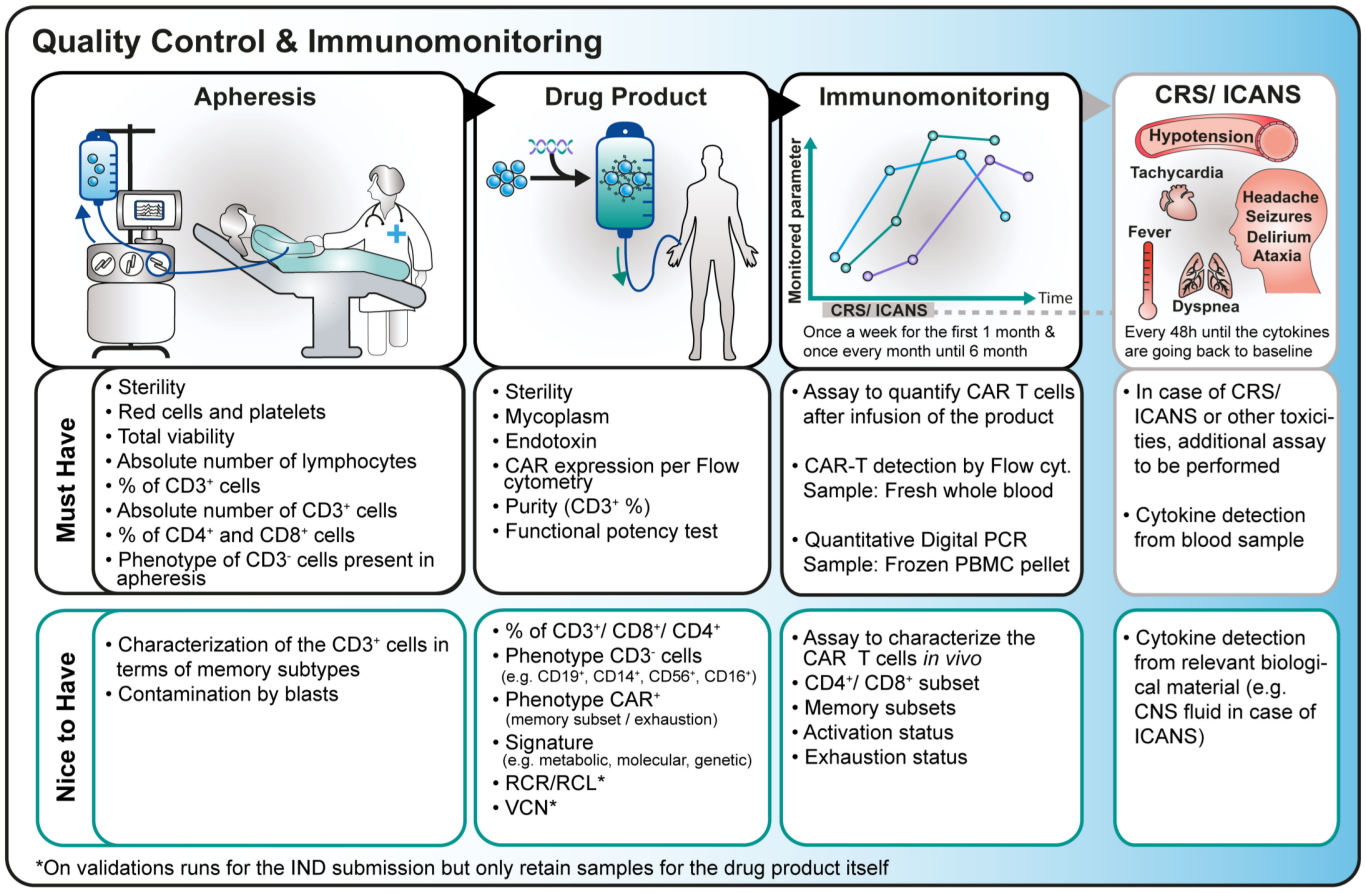
Overview of the current practice and ideal “nice-to-have” scenario of assays intended for apheresis, drug product and immunomonitoring characterization.

Overall, the significant heterogeneity in the daily practices of leukapheresis characterization, CAR T-cell product analysis, and patient immunomonitoring strategies highlights the urgent need for standardization to achieve an ideal “nice-to-have” scenario in this field (**Figure 7**), enhancing drug product characterization and improving patient management. Establishing standardized practices would benefit both patients and healthcare professionals by ensuring more reliable results and generating robust data to guide clinical decision-making.

## Data Availability

The original contributions presented in the study are included in the article/**Supplementary Material**. Further inquiries can be directed to the corresponding author/s.

## Supplementary material

Supplementary Table 1 Structured sections of the survey. This table outlines the various sections that comprise the T2Evolve survey, providing an overview of its structure and organization.

Supplementary Table 2 Tabular view of the survey questions. This table lists the questions included in the T2Evolve survey, organized into sections (Section A through Section F) to provide a detailed breakdown of the survey’s structure.

Supplementary Table 3 Distribution of Survey Respondents by European country and city. This table presents the number and percentage of survey European respondents, organized by country and city, offering a detailed geographic distribution.
