# Supplementary material for: European survey on CAR T-Cell analytical methods from apheresis to post-infusion immunomonitoring
Source: Front Immunol. 2025 Apr 24;16:1567582. doi: 10.3389/fimmu.2025.1567582 (PMC12058815; doi:10.3389/fimmu.2025.1567582)
Supplement: Supplementary Table 1 — Structured sections of the survey. This table outlines the various sections that comprise the T2Evolve survey, providing an overview of its structure and organization. [file DataSheet1.docx]

**Table S1**

| **T2EVOLVE Survey​​ is organized in six different sections (44 questions)** | |
| --- | --- |
| **Section** | **Title** |
| A | Work Information |
| B | Technical information about manufacturing and quality controls (QC) of apheresis product |
| C | Technical information on QC of raw materials used for the manufacturing of engineered T-cells |
| D | Technical information about manufacturing and QC of engineered T-cells final product (FP) and Release criteria |
| E | Technical information about Lympho-depletion regimen |
| F | Technical information about Immuno-monitoring plan |

**Table S1. Structured sections of the survey.** This table outlines the various sections that comprise the T2Evolve survey, providing an overview of its structure and organization.

**Table S2**

| **T2EVOLVE Survey​​ Section A** | |
| --- | --- |
| **Number​​** | **Question​s about work Information​** |
| N1​​ | In what country do you work?​​ |
| N2​​ | In what city do you work?​​ |
| N3​​ | In what type of organization do you work?​​ |
| N4​​ | In which field/department do you work​? Clinical (oncology, pediatric, hematology, other,…)​ |
| N5​ | Which indication(s) and population are you/your department mostly working with/for? ​You can select multiple answers: ​ |
| N6 | In which situations are you/your organization administering engineered T-cells? |
|  | |
| **T2EVOLVE Survey​​ Section B** | |
| **Number​** | **Questions about manufacturing and QC of apheresis product​** |
| N1​ | Do you/your organization perform/request analysis on apheresis product intended for the manufacturing of engineered T-cells products?​ |
| N2​ | Which assay do you perform/request on apheresis product (fresh or post-thaw) intended for the manufacturing of autologous engineered T-cells products?​ |
| N3​ | Except from compatibility/HLA tests, which assay do you Perform/request on apheresis product (fresh or post-thaw) intended for the manufacturing of allogeneic engineered T-cells products?​ |
| N4​ | According to your perspective and expertise, which analytical control(s) are critical and thus should be performed before use of an apheresis product (fresh or post-thaw) in engineered T-cells manufacturing?​ |
| N5 | Based on your experience, what could be improved in the analytical control of apheresis product to standardize practices? |
|  | |
| **T2EVOLVE Survey​​ Section C** | |
| **Number​** | **Question​s about QC of raw materials used for the manufacturing of engineered T-cells​** |
| N1​ | Do you/your organization perform any analytical testing on raw materials (for release, stability study or other purpose) used for the manufacturing of engineered T-cells-cells, as per viral vector, plasmid DNA, RNA, transposon/transposase, CRISPR Cas 9?​ |
| N2​ | Which gene transfer material are you/your organization mostly using to manufacture engineered T-cells product?​ |
| N3​ | Which quality attribute(s) of this material are you/your organization verifying at your GMP facility (initially or in addition to the Documentation/certificate of analysis provided by the manufacturer)?​ |
|  | |
| **T2EVOLVE Survey​​ Section D** | |
| **Number​** | **Questions about manufacturing and QC of engineered T-cells FP and Release criteria​** |
| N1​ | Are you/your organization involved in the manufacturing/release of an engineered T-cell final product?​ |
| N2​ | Which assay do you perform (for information, release or stability study) on autologous Final Product?​ |
| N3​ | Which assay do you perform (for information, release or stability study) on allogeneic Final Product?​ |
| N4​ | According to your perspective, which assay(s) is/are critical for the release of an engineered T-cell product?​ |
| N5 | Based on your experience, what would be the major needs in terms of standardization regarding the release of engineered T-cells products? |
|  | |
| **T2EVOLVE Survey​​ Section E** | |
| **Number​** | **Questions about Lympho-depletion regimen** |
| N1​ | Which lymphodepletion regimen do you have experience with?​ |
| N2​ | How do you characterize patient's samples collected before lymphodepletion?​ |
| N3​ | How do you characterize blood samples from patients after the lymphodepletion but before engineered T-cells infusion?​ |
| N4​ | Based on your experience, what would be the major needs in terms of standardization regarding pre-infusion monitoring and/or lymphodepletion? |
|  | |
| **T2EVOLVE Survey​​ Section F** | |
| **Number​** | **Questions about Immuno-monitoring plan​** |
| N1​ | According to your practice, which assay do you generally request/perform to quantify engineered T-cells after infusion of the product?​ |
| N2​ | According to your practice, which assay is critical to characterize engineered T-cells after infusion of the product?​ |
| N3​ | Do you perform analysis of engineered T-cells from other types of samples than blood?​ |
| N4​ | In case of CRS1/ICANS2 or other toxicities, do you perform further analytical measures?​ |
| N5 | In case of relapse, do you perform further analytical measures?​ |
| N6 | Do you perform immunogenicity of engineered T-cells?​ |
| N7 | After infusion of which type of product do you have experience of post-infusion monitoring (characterization and quantification of engineered T-cells)?​ |
| N8​ | Frequency of the immuno-monitoring:​ |
| N9​ | Duration of the immuno-monitoring:​ |
| N10 | Following the occurrence of CRS/ICANS/other toxicities (after infusion of CAR-T cells), do you perform additional immuno-monitoring?​ |
| N11 | Frequency of the additional immuno-monitoring:​ |
| N12 | Duration of the additional immuno-monitoring:​ |
| N13 | Based on your experience, what would be the major improvements needed for a more relevant and efficient immuno-monitoring of patients in the first 12 months following engineered T-cells infusion? |

**Table S2. Tabular view of the survey questions.** This table lists the questions included in the T2Evolve survey, organized into sections (Section A through Section F) to provide a detailed breakdown of the survey’s structure.

**Table S3**

|  |  |  |  |  |
| --- | --- | --- | --- | --- |
|  | **Country names** *City names* | Number of respondents | % of respondents among all |  |
|  | **Italy** | **16** | **30.19** |  |
|  | *Rome* | 9 |  |  |
|  | *Milano* | 3 |  |  |
|  | *Bologna* | 2 |  |  |
|  | *Verona* | 1 |  |  |
|  | *Meldola* | 1 |  |  |
|  | **France** | **9** | **16.98** |  |
|  | *Paris* | 6 |  |  |
|  | *Montpellier* | 1 |  |  |
|  | *Marseille* | 1 |  |  |
|  | *Bouffere* | 1 |  |  |
|  | **Germany** | **9** | **16.98** |  |
|  | *Bergisch Gladbach* | 4 |  |  |
|  | *Leipzig* | 2 |  |  |
|  | *Würzburg* | 1 |  |  |
|  | *Mainz* | 1 |  |  |
|  | *Frankfurt* | 1 |  |  |
|  | **Czechia** | **4** | **7.55** |  |
|  | *Prague* | 1 |  |  |
|  | *Ostrava* | 1 |  |  |
|  | *Hradec Králové* | 1 |  |  |
|  | *Brno* | 1 |  |  |
|  | **Spain** | **3** | **5.66** |  |
|  | *Valencia* | 1 |  |  |
|  | *Salamanca* | 1 |  |  |
|  | *Barcelona* | 1 |  |  |
|  | **Netherlands** | **3** | **5.66** |  |
|  | *Amsterdam* | 2 |  |  |
|  | *Nijmegen* | 1 |  |  |
|  | **Austria** | **2** | **3.77** |  |
|  | *Vienna* | 1 |  |  |
|  | *Innsbruck* | 1 |  |  |
|  | **Finland** | **2** | **3.77** |  |
|  | *Oulu* | 1 |  |  |
|  | *Helsinki* | 1 |  |  |
|  | **United Kingdom** | **1** | **1.89** |  |
|  | *London* | 1 |  |  |
|  | **Norway** | **1** | **1.89** |  |
|  | *Bergen* | 1 |  |  |
|  | **Greece** | **1** | **1.89** |  |
|  | *Athens* | 1 |  |  |
|  | **Slovenia** | **1** | **1.89** |  |
|  | *Ljubljana* | 1 |  |  |
|  | **Croatia** | **1** | **1.89** |  |
|  | *Zagreb* | 1 |  |  |
|  |  |  |  |  |
|  |  |  |  |  |

**Table S3.** Distribution of Survey Respondents by European country and city. This table presents the number and percentage of survey European respondents, organized by country and city, offering a detailed geographic distribution
